# Monthly Summary

**Published:** 1885-11

**Authors:** 


### Monthly Summary.

A PECULIAR CASE.—John Thompson was ill with malarial fever, and I prescribed for him Quinia Sulphas. The most peculiar circumstance about this case was as follows; John, as a matter of fact, had to remain in the house, and as he had nothing else to do, he held their ten-months-old baby on his lap a considerable portion of the time. During the four weeks that John was sick, the baby had four teeth peep through the gums. The question at once arose in my mind, how did the quinine act to produce this result? After making a few circus rings around my bump of superlativeness, I evolved the following attenuated explanation. Naturally the child would inhale more or less of John's breath. John was taking the medicine, and of course he had a quinine-laden respiration. The child inhaled this, the quinine-germs came into contact with the gums and produced this evolution of the teeth. Or, to be more explicit, the teeth are composed of a substance that has sufficient density to produce a solution of continuity of a softer structure. The quinine-germs have a peculiar elective affinity for the teeth of children, they being rounded and located *subgumma*. The quinine-germs, being attracted by the magnetism that flows from the teeth to the food to assist in preparing it for the circulation of the liver, accumulate upon the gums over the tooth. In union there is strength, and as these germs increase in number at this place they form a powerful battery, and draw the tooth to a point, and the point pierces the solution of continuity in the gum. In the solution of this heretofore unexplained mystery we have made a wonderful discovery, and wish to call the attention of the faculty to this seeming *rara avis*.—*Cincinnati Lancet and Clinic.*

OXY-PHOSPHATE.—*By E. G. Betty, D. D. S., Cincinnati.* —Under the head of "Different Materials for, and Methods of Filling Teeth," it may not be amiss to say a few words about our very good servant, oxy-phosphate of zinc.

This substance, as all are aware, is the result of a chemical union between a base and an acid; but with that part of it we will have nothing to do to-day. I am daily becoming more convinced that this material is of more service than we imagine, in this, that it enables us to ultimately save more "aching" teeth than we could were we still dependent upon oxychlorides. It is my custom to fill *all* teeth with it in which the decay has been so extensive as to nearly expose the pulp, those in which it *is* exposed, and dead teeth that have undergone conservative treatment. In case the decay has not exposed the pulp, the debris is thoroughly removed and the cavity filled with a bolus of the phosphate mixed as dry as possible; the surplus is then removed and the filling covered with wax or paraffin, or it may be given a smooth surface by polishing with heated talc. Should the pulp be exposed, remove the decay, cover the pulp with some kind of varnish, (comp. tinct. of benzoin is most excellent), flow over it a little of the phosphate prepared very thin; when this has hardened, trim and fill as in the first case. Dead teeth are also to be filled with stiff bolus. The main point in the use of the phosphate, if you wish to secure full service from it, is to allow the filling to remain as long as it will last, which, in very many cases, is about two years. Should it not remain so long, refill. The object of this is to give the tooth a *long rest*, the longer the better at the end of a year and a half or two years; large operations with cohesive foil will be borne by the tooth so treated with but a slight chance of producing either death of the pulp in the one case or recurrence of periosteal inflammation in the other.

A writer in the *Independent Practitioner* for May, recommends that a small quantity of iodoform be mixed with the thin oxy-phosphate to be flowed on exposed pulps, or immediately over the thin dentine covering one almost exposed. Though I have not tried this, yet I am inclined to think the principle is good.—*Ohio State Journal of Dental Science.*

